# Management of Nipple Necrosis and Wound Complications in Patients Undergoing Unilateral Skin-Sparing Mastectomy and Implant-Based Reconstruction for Breast Cancer: A Retrospective, Single-Center Study

**DOI:** 10.3390/medicina62030575

**Published:** 2026-03-19

**Authors:** Simay Akyuz, Şevket Barış Morkavuk, Mehmet Ali Gülçelik

**Affiliations:** 1 Department of Oncology Nursing, Gulhane Faculty of Nursing, University of Health Sciences, 06020 Ankara, Turkey; 2 Department of Surgical Oncology, Gulhane Research and Training Hospital, University of Health Sciences, 06020 Ankara, Turkey; drsbmor@yahoo.com (Ş.B.M.); mgulcelik@yahoo.com (M.A.G.)

**Keywords:** breast cancer, skin-sparing mastectomy, implant-based breast reconstruction, nipple–areola complex necrosis, wound dehiscence, postoperative complications, wound management

## Abstract

*Background and Objectives:* The aim of this retrospective cohort study was to determine the frequency of early-stage nipple necrosis and wound complications in patients undergoing unilateral skin-sparing mastectomy (SSM) and direct implant-based reconstruction and describe the conservative/advanced wound care approaches used for these complications. *Materials and Methods:* A retrospective review was made of the medical records of 84 patients who underwent same-session unilateral SSM and implant-based reconstruction in the Surgical Oncology Clinic between November 2019 and February 2024. Statistical analyses were performed using the Shapiro–Wilk test, Mann–Whitney U-test, and Chi-square/Fisher tests. *Results*: The mean age of the patients was 43.51 ± 6.5 years, 35.7% of the patients received neoadjuvant chemotherapy, and smoking prevalence was 7.1%. Wound complications developed in 16.7% of the patients, distributed as follows: wound dehiscence 6%, NAC necrosis 8.4%, infection 1.2%, and hematoma 1.2%. Interventions due to complications were performed at rates of 2.4% for areola excision, 2.4% for debridement, and 2.4% for implant excision. The only variable significantly associated with complication development was excision volume, which was higher in the complication group (*p* = 0.033). Logistic regression analysis showed that a one-unit increase in excision volume was associated with a statistically significant increase in the likelihood of complication development (O.R = 1.002; 95% CI: 1.000–1.004; *p* = 0.019). No significant association was found between age, height/weight, neoadjuvant therapy, smoking, breast side, pathology subtype, axillary approach, and the development of complications (*p* > 0.05). Advanced wound management was provided in 10 of the 14 cases (71.4%) that developed complications. *Conclusions*: Excision volume was found to be the only variable associated with wound complication development after implant-based reconstruction following unilateral SSM. Most complications were managed successfully with advanced wound care, minimizing the need for re-operation. For patients undergoing high-volume excision, risk-based early multidisciplinary, close follow-up is recommended.

## 1. Introduction

Breast cancer is a prevalent malignancy among women worldwide in terms of both incidence and mortality [1]. Since the 1990s, the widespread use of mammography and introduction of effective systemic therapies such as tamoxifen have contributed to decreased mortality rates [2]. Advances in screening and therapeutic strategies have increased the five-year survival rate for breast cancer to over 80% [3].

The classic surgical treatment for breast cancer is mastectomy, and in the classic mastectomy approach, the nipple–areola complex (NAC) is removed in addition to the entire breast tissue [4]. However, breast-conserving surgery is now more frequently preferred due to comparable oncological outcomes, lower morbidity, and positive effects on quality of life [5].

In patients for whom breast-conserving surgery is not appropriate, implant-based breast reconstruction after skin-sparing mastectomy (SSM) is an important option. The oncological safety of SSM has been proven to have recurrence rates equivalent to those after standard total mastectomy, and it is accepted as a standard mastectomy procedure without increasing the risk of local recurrence [6]. While disease control is the primary aim of treatment with this surgical technique, restoring the breast shape after mastectomy has been reported to have positive effects by alleviating the physical and emotional trauma experienced by patients [7]. Furthermore, studies have indicated that patients who undergo nipple-sparing and implant-based reconstruction report higher satisfaction, better psychosocial well-being, and improved sexual well-being [8,9].

Implant-based reconstruction after mastectomy can be performed in a single stage (direct implant) or in two stages (tissue expander followed by implant) [10]. Skin-sparing mastectomy aims for a more natural esthetic appearance compared to standard mastectomy by preserving a large portion of the breast skin cover [4]. Preserving the natural skin cover allows the surgeon to reconstruct the breast with a permanent implant in the same session (direct implant), thereby eliminating the need for tissue expansion [11].

However, as with any surgical procedure, postoperative complications and the need for re-operation are undesirable. Complications not only negatively affect cosmetic results and costs; more importantly, they can delay adjuvant treatments [12]. There are reports in the literature of major/minor complications such as bleeding, surgical site infection, seroma, hematoma, wound dehiscence, and necrosis following skin-sparing mastectomy and implant-based reconstruction [13]. Among these complications, NAC necrosis and wound dehiscence stand out as problems that are both difficult to manage clinically and have a high potential for requiring re-operation [10]. Necrosis and wound dehiscence are closely related to tissue perfusion, flap thickness, and surgical technique. Previous studies have reported a skin flap necrosis rate of up to 30% in patients with a 4–5 mm thick skin flap after skin-sparing mastectomy [14]. It has also been suggested that neoadjuvant chemotherapy and radiotherapy may increase the risk of postoperative necrosis and wound dehiscence [13]. Therefore, re-operation may be necessary if NAC necrosis or wound dehiscence develops. As the early detection and appropriate management of NAC necrosis and wound complications that may develop early in this patient group are critical to be able to reduce the need for re-operation and avoid delays in adjuvant treatments, patients should be closely monitored by a multidisciplinary team that includes experienced wound care professionals.

The aim of this retrospective cohort study was to determine the frequency of early-stage NAC necrosis and wound complications in patients who underwent unilateral SSM and direct implant-based reconstruction and describe the conservative/advanced wound care approaches used when these complications developed.

## 2. Methods

This retrospective, single-center cohort analysis was conducted in the Surgical Oncology Clinic of a Training and Research Hospital.

### 2.1. Patient Selection

Patients who underwent a mastectomy and implant procedure due to breast cancer diagnosis between November 2019 and February 2024 were screened. A total of 180 patients who underwent bilateral mastectomy and prosthesis were identified. After the exclusion of 61 patients who underwent autologous reconstruction, 4 patients with missing records, and 31 for other reasons, 84 patients who underwent unilateral SSM + direct implant were included in this study.

### 2.2. Inclusion Criteria

1. Patients who underwent unilateral skin-sparing mastectomy (SSM) during the specified time period and underwent direct (single-stage) implant-based reconstruction during the same session.
2. Patients for whom postoperative clinical follow-up or complication records were available.

### 2.3. Exclusion Criteria

1. Patients who underwent prophylactic bilateral mastectomy and implant-based reconstruction due to high-penetration gene mutations (e.g., BRCA1, BRCA2, TP53, CDH1, PTEN).
2. Patients who underwent prophylactic bilateral mastectomy and implant-based reconstruction due to a strong family history in addition to a low-penetration gene mutation.

### 2.4. Surgical Technique

In this study, all the mastectomies were performed by oncological surgeons with similar levels of experience to minimize surgeon-related variability, and as far as possible, to control for differences in mastectomy technique and skin flap quality. All patients included in the study underwent subpectoral implant placement via radial incision. No supramuscular or hybrid implant placement was performed. In all patients, areola and skin flap perfusion adequacy was evaluated using indocyanine green angiography (ICGA) after implantation. In this method, microvascular perfusion of the areola and skin flap is evaluated by applying indocyanine green after implantation. In patients where inadequate perfusion was detected in the nipple–areola complex as a result of this evaluation, which is routinely used in our clinic, areola resection was performed. No problems were detected in any of the study cases in nipple–areola and skin flap perfusion in the ICGA evaluation.

### 2.5. Postoperative Nipple Necrosis and Wound Complication Classifications

Early wound complications were defined as complications occurring within the first 30 days postoperatively. All complications were confirmed through surgical examination and clinical records (photographic records and/or wound care assessment notes) in the patient’s file.

NAC necrosis depth was classified according to the depth component definitions of the SKIN scoring system [15]. NAC ischemia/necrosis was clinically assessed as discoloration and decreased capillary refill. NAC necrosis grading was performed using file and photograph-based records.

- Partial NAC necrosis: Superficial or limited full-thickness tissue loss in part of the areola and/or nipple.
- Complete NAC necrosis: Findings consistent with full-thickness tissue loss/scarring in the entire NAC, and where possible, the necrosis depth was noted as “superficial (only epidermis/dermis)” or “full-thickness”; if records did not include this distinction, it was reported as “unspecified”.

Wound dehiscence: Wound dehiscence was defined as a disruption of skin integrity at the incision line (≥5 mm gap or recorded as “dehiscence” by the clinician) requiring additional dressing/advanced wound care or surgical intervention.

Surgical site infection: Surgical site infection (SSI) was defined in accordance with the CDC/NHSN SSI criteria as a clinically diagnosed infection characterized by erythema and/or increased local warmth, purulent drainage, malodor, tenderness, and/or systemic manifestations, with documentation of the initiation of antibiotic therapy and/or a wound culture [16].

Hematoma: This was defined as the detection of a collection on clinical examination, and/or confirmation via ultrasound, and/or requiring aspiration/drainage/surgery.

### 2.6. Postoperative Nipple Necrosis and Wound Complication Management

The product selection for advanced wound care was based on the wound bed. Treatment was initiated on the postoperative day when the complication was first identified by the surgeon. The dressing change frequency was adjusted to daily or every 48–72 h based on the exudate volume and necrosis level. In addition, hyperbaric oxygen therapy (HBOT) was applied to support wound management in patients where needed and appropriate to the indication. In this adjunctive technique, the aim is to increase tissue oxygenation with HBOT, prevent ischemia–reperfusion injury, reduce edema, and promote neovascularization. In this way, the progression of ischemia to necrosis can be prevented and necrosis can be limited. The HBOT treatment protocol consisted of 2 h sessions for 30 days. The objective endpoint of healing was defined as the day when complete epithelialization was achieved. Cases with complications were followed-up until complete epithelialization was achieved.

### 2.7. Establishment of the Variables’ and Specimens’ Excisional Volume

The following variables were analyzed to evaluate etiopathological factors that may be associated with the development of necrosis and wound complications and to create an algorithm for the management of these factors: demographic parameters (age, height, weight), smoking history, breast side, histopathological data, and surgical criteria including axillary approach and excisional volume.

Excision volume was calculated based on the three dimensions (in cm) of the surgical specimen, as measured by the pathologist. Assuming that the specimen is macroscopically ellipsoidal, the excisional volume formula [4/3π(a*b*c)] was used as previous studies have shown it to provide a standardized volume estimate [17,18]. In this formula, a, b, and c represent halves (half-axes) of the measured length–width–depth values. Thus, the excisional volume of all the specimens in this study was determined using a standard method.

### 2.8. Ethical Considerations

Approval for this study was granted by the Local Ethics Committee (decision no: 2025-490, dated: 21 October 2025).

### 2.9. Statistical Analysis

Data obtained in the study were analyzed statistically using SPSS for Windows, vn. 31.0.1.0 software (IBM Corporation, Armonk, NY, USA). The normality of the data distributions was assessed using the Shapiro–Wilk test, and the Levene test was used to examine heterogeneity. Age and weight were seen to follow normal distribution, whereas height and excision volume did not. Accordingly, both parametric and non-parametric tests were used. Descriptive statistics are presented as mean ± standard deviation (SD) values for continuous data, and as number (n) and percentage (%) for categorical variables. In the comparisons of categorical variables, Pearson’s Chi-square test was used, with Fisher’s Exact or the Fisher–Freeman–Halton Exact test when the expected cell counts were insufficient. The independent-samples *t*-test was applied to make comparisons of continuous variables with normal distribution, and the Mann–Whitney U-test was used when distribution was not normal. Univariate logistic regression analysis was performed to determine potential outcome determinants. The results were reported as odds ratios (ORs) and 95% confidence intervals (CIs). A value of *p* < 0.05 was accepted as statistically significant.

## 3. Results

### 3.1. Patient Characteristics

A total of 84 patients who underwent direct implant-based breast reconstruction after unilateral skin-sparing mastectomy were analyzed. The mean age of the patients was 43.51 ± 6.5, 7.1% were smokers, and 15.5% had chronic diseases. In 58.3% of cases, the tumor was located in the right breast, and 51.2% had multicentric–multifocal involvement. The histopathological diagnosis was invasive ductal carcinoma in 66.7% of the patients, and neoadjuvant chemotherapy was administered to 35.7%. The mean excision volume was determined to be 521.45 ± 290.92 cm^3^. In axillary management, SLNB was performed in 77.3% of patients and ALND in 22.6%. The demographic and clinicopathological distributions of all patients who underwent implant-based reconstruction after unilateral SSM are presented in Table 1.

### 3.2. Evaluation of Wound Complications

Wound complications developed in 14 (16.7%) of the 84 patients included in this study. When the wound complications that developed were examined, wound dehiscence was found in 6%, nipple necrosis in 8.4%, wound infection in 1.2%, and hematoma in 1.2%. To treat these complications, areola excision was performed in 2.4% of patients, wound debridement in 2.4%, and implant excision in 2.4%. In 10 of the 14 cases (71.4%) with wound complications, epithelialization and wound healing were achieved through conservative wound care using advanced wound care products (Table 1).

### 3.3. Distribution of Clinical–Pathological Factors According to Wound Complication Groups

Of the 84 patients included in the study, 14 developed wound complications, while 70 showed no complications. When comparing the groups with and without complications, no significant differences were found in terms of age, height, and weight (*p* > 0.05 for all). No statistically significant relationship was seen between smoking, breast side, pathology subtype, axillary surgical approach, and neoadjuvant chemotherapy with the development of wound complications (*p* = 0.388, *p* = 0.921, *p* = 0.136, *p* = 0.652, *p* = 0.541, respectively). The distributions of clinical–pathological characteristics are presented in Table 2.

### 3.4. Relationships Between Excision Volume and Wound Complications

A significant relationship was determined between excision volume and the development of wound complications. Excision volume was higher in patients who developed complications (U = 294, *p* = 0.033); the median excision volume was found to be 438.0 cm^3^ in the group without complications and 637.5 cm^3^ in the group with complications (Table 2). This finding was confirmed with univariate logistic regression analysis (OR = 1.002; 95% CI: 1.000–1.004; *p* = 0.019). An increase in excision volume as seen to increase the probability of complications; the accuracy rate of the model was 83.8% (Table 3).

### 3.5. Management of Wound Complications

**Scheme 1:**
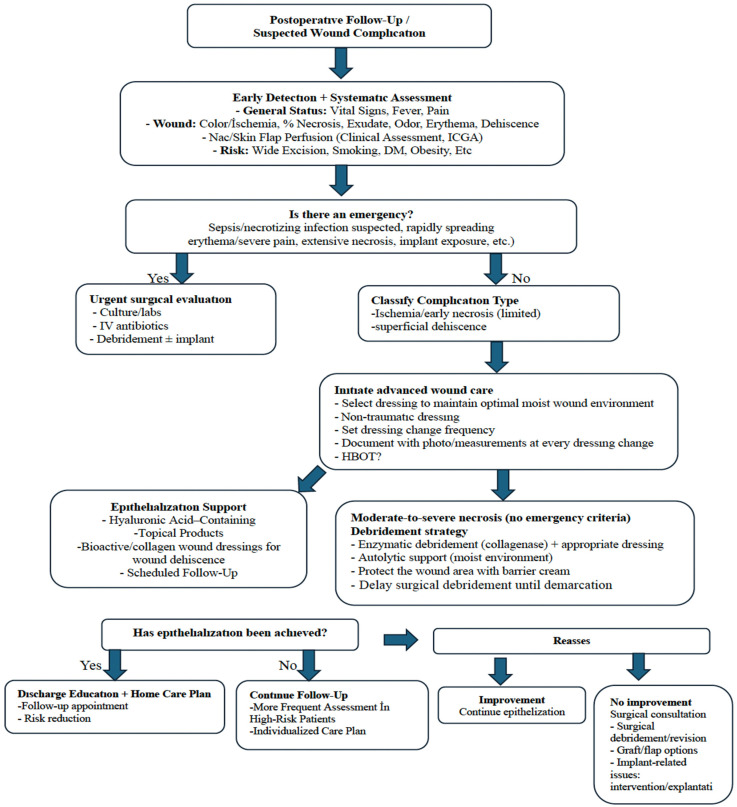
Wound care algorithm clinical experience flowchart. ICGA, indocyanine green angiography; DM, diabetes mellitus; HBOT, hyperbaric oxygen therapy.

The methods used in advanced wound care for 10 of the 14 cases (71.4%) that developed complications are summarized in Table 4 and Scheme 1. Four of these patients were monitored for wound dehiscence, five for NAC necrosis, and one for wound infection. Since the clinical characteristics of each wound can vary (wound bed, extent/depth of necrosis, signs of infection, periwound skin integrity, etc.), the routine use of a single product for all cases is not appropriate. Therefore, in the selection of materials and modalities for use in advanced wound care, there should be consideration of the condition of the wound, the targeted care objective (debridement, maintaining moisture balance, bioburden control, supporting granulation, accelerating epithelialization, etc.), and patient-specific accompanying factors (comorbidities, smoking history, nutritional status). The wound care approach implemented in this study was based on an “assess–reassess” algorithm. At each dressing change, the wound bed (necrotic/slough/granulation/epithelializing), signs of infection, and the condition of the periwound skin were evaluated, and the wound care plan was updated accordingly. Given the dynamic nature of necrosis, wound status was reassessed on average every 48–72 h, and wound care products were modified when deemed necessary. Non-traumatic dressings were used in patients who developed complications. This is a fundamental practice to ensure optimal moisture balance in the wound bed and to prevent dressings such as gauze from adhering to the wound bed. It also provides the advantage of protecting the wound bed from minor trauma with each dressing change. Selection of an atraumatic dressing consisted of dressings incorporating a non-adherent hydropolymer contact layer, and depending on the level of exudate, may require reinforcement with a secondary dressing. In some cases, the sequential or combined use of different wound care products (e.g., approaches targeting both ongoing debridement and epithelialization) was required. In patients with partial necrosis, enzymatic debridement gel is used to resolve the necrosis, while in granular wound areas, products that contribute to epithelialization are used simultaneously. In the current study cases, enzymatic debridement gels were applied as a thin layer to areas containing necrotic/slough tissue and were monitored with dressing changes performed every 48 h. To prevent irritation of the intact periwound skin, a barrier product was applied to the periwound area to confine the debridement effect to the target tissue. Slough/necrotic tissue was gradually removed from the wound bed, starting at the margins, using gentle sharp debridement. During the concurrent epithelialization phase, topical agents containing hyaluronic acid were preferred to protect granulation tissue and support new epithelial formation; these agents were used in combination with atraumatic dressings to maintain the principle of moist wound healing. As wounds epithelialize starting from the edges, protecting the periwound tissue is crucial for the progression of epithelialization. Therefore, products that support the skin barrier were used around areas where necrosis and wound dehiscence were observed. Hyperbaric oxygen therapy was applied to three patients (two who developed partial NAC necrosis and one who developed wound dehiscence) to support wound management. The HBOT protocol consisted of 2 h sessions for 30 days. In patients who developed complications, the standardization of the wound care approach was based on an assessment–reassessment algorithm. When the epithelialization time of the patients was evaluated, the median (IQR) was found to be 53 (43) days, and no patient returned due to wound recurrence or wound complications during the follow-up period in our Surgical Oncology Clinic.

## 4. Discussion

The results of this study showed that 16.8% of patients who underwent unilateral SSM and direct implant-based reconstruction in the same session developed nipple necrosis and/or wound complications, and 71.4% of the complications that occurred were successfully managed with conservative wound care. A statistically significant relationship between the development of complications and excision volume was seen, indicating that the development of complications may increase as the excision volume increases.

The literature indicates that the overall complication rate in reconstructions performed with direct implant placement ranges from 24.1% to 30.3%, with the most frequently reported complications being skin flap necrosis, delayed wound healing, infection, seroma, and hematoma [19,20,21]. The lower complication rate observed in the current study compared to the range reported in the literature may be related to patient selection, differences in surgical techniques, and variability in perioperative management protocols. Furthermore, it can be considered that clinical experience with SSM and direct implant-based reconstruction, together with a standardized perioperative care/monitoring approach, may have contributed to the prevention of complications, and when they did occur, to their control through early conservative management.

In this study, age, anthropometric measurements (height, weight/BMI), smoking, tumor characteristics (location, side, pathological subtype), axillary surgical approach, and neoadjuvant treatment variables did not show a statistically significant relationship with the development of complications. Recent studies have revealed heterogeneous findings regarding variables affecting the risk of complications. One study emphasized that the incidence of complications may increase in patients over 50 years of age, which could be associated with age-related dermal thinning, but no significant association with smoking was found [22]. In contrast, another study evaluating breast reconstructions performed after neoadjuvant chemotherapy found that high BMI and tobacco use were associated with complications [23]. Another study indicated that patient weight and breast resection weight were significant risk factors for skin flap necrosis and NAC necrosis, while tumor localization was not associated. Although smoking history and primary systemic treatment (including neoadjuvant chemotherapy) are theoretically predicted to negatively affect tissue perfusion and lead to ischemic changes, some studies have failed to demonstrate a statistically significant effect of these variables on skin flap necrosis [24]. The differences between the current study’s results and those in the literature may be due to methodological differences in aspects such as the sample size, patient selection, surgical technique/team’s experience, and definitions of complications. In this context, the results of this study indicate that complications after SSM have a multifactorial structure and that larger, well-designed studies are needed to more clearly identify risk factors.

In the current study, the only variable that showed a significant association with the development of complications was the excision volume. According to these findings, the probability of developing complications increases as the excision volume increases. The literature has also indicated that the risk of complications may increase as the weight/volume of tissue removed via mastectomy increases [11]. It has been suggested that higher excision volumes may reduce perfusion in mastectomy flaps, thereby increasing the risk of flap necrosis and delayed wound healing, and that increased surgical tissue trauma and dead space, together with impaired lymphatic drainage, may further adversely affect wound healing [25]. Therefore, during preoperative planning, anticipating the mastectomy volume and the expected flap thickness and adopting an approach focused on minimizing dead space, reducing closure tension, and preserving flap/NAC perfusion may help decrease the risk of early postoperative complications. Although mastectomy volume has been reported to be associated with necrosis, it should also be noted that this association has not been consistently confirmed as an independent risk factor in multivariable analyses [26]. Moreover, the role of biomarkers in predicting complication risk is also being investigated. For example, it has been suggested that butyrylcholinesterase (BChE) levels may be associated with the risk and severity of postoperative infection [27]. Although the role of predictive/diagnostic biomarkers in wound complications after breast cancer surgery is not yet clear, it has been emphasized that some biomarkers associated with chronic wound healing have been identified and that this area requires further study [28].

In managing risks associated with increased excision volumes, intraoperative perfusion assessment and appropriate incision selection appear to be critical. It has been reported that the risk of post-reconstruction necrosis may be related to blood flow and the use of a periareolar incision. More specifically, periareolar incisions may adversely affect perinipple perfusion, and therefore preservation of intercostal perforating vessels is considered important [26,29]. Accordingly, it should be taken into account that incision choice and tissue trauma secondary to an expanded surgical field may compromise perforator vessels and thereby reduce local perfusion. In the current study, consistent with the literature, intraoperative microvascular/perfusion assessment was performed in all patients using ICGA. However, it should be kept in mind that ICGA provides an intraoperative, timepoint assessment, and that perfusion may subsequently deteriorate due to postoperative edema, hematoma/seroma-related compression, external compression, or implant-related tension. In addition, traction on the NAC during sub-nipple resection may increase microtrauma and edema, potentially contributing to delayed ischemic changes.

In the postoperative period, close clinical monitoring, assessment of early perfusion/ischemia signs, appropriate dressing management and avoidance of compression, rapid intervention in cases of suspected seroma/hematoma, and prompt initiation of treatment in cases showing signs of infection can be crucial in preventing complications and progression.

The prominence of NAC necrosis and wound dehiscence in the complication distribution is an expected finding in cases where the perfusion of the mastectomy skin flap after SSM may be limited. It has been reported in the literature that excision volume [24] and increased tension caused by the implant, particularly in large and sagging breasts, are the main factors contributing to the development of necrosis due to impaired flap perfusion associated with mastectomy [20,30]. Although the complication profile in the current study was consistent with this mechanism, the need for re-operation was low, and only a few patients underwent areola excision, debridement, or implant excision.

The fact that epithelialization was achieved with advanced wound care in 71.4% of the current study patients who developed complications suggests that conservative wound management in appropriate patients may reduce the need for surgical intervention. Monitoring the epithelialization period as 53 (43) days in patients with complications supports that most wound complications can be managed without the need for re-operation or unplanned re-admission in most patients with structured conservative/advanced wound care and close monitoring. Given the limited availability of data quantitatively reporting time to epithelialization, this finding may provide a practical reference for patient counselling and follow-up planning. Although this duration was observed to cause only a minimal delay in the initiation of adjuvant therapy, it underscores the clinical importance of implementing appropriate wound management algorithms to optimize outcomes.

**Figure 1:**
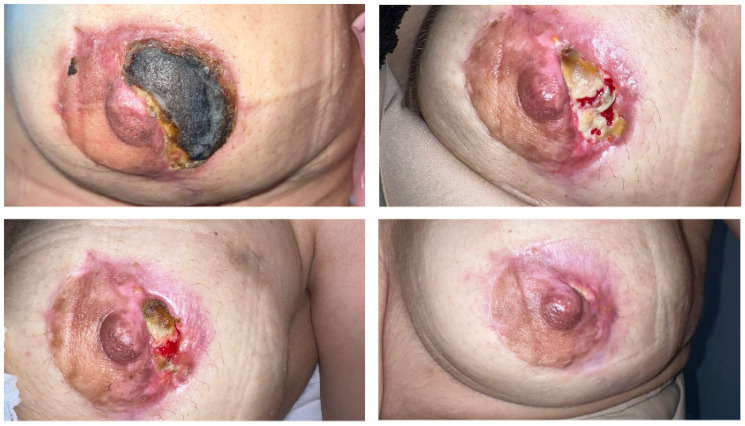
Example case managed with the wound care algorithm.

In our clinic, advanced wound management is carried out by an experienced wound care nurse and team with structured follow-up; the tissue composition of the wound bed (necrosis/slough/granulation/epithelialization), surrounding skin integrity (maceration/erythema), pain, and signs of infection are systematically evaluated during daily or regular check-ups, and the dressing selection and change frequency are adjusted according to these parameters. In line with the principles of moist wound healing, the goal is to maintain optimal moisture balance with dressings appropriate to the exudate level. In this context, collagenase-containing enzymatic debridement gels are used to remove necrotic tissue, and topical products containing hyaluronic acid are used during the epithelialization phase. Furthermore, it is considered appropriate to prefer non-traumatic dressings, avoid unnecessary frequent dressing changes, and limit compression applications that may negatively affect perfusion. Although the aim is to standardize a general approach to wound and NAC necrosis management, individualized care is essential. The monitoring frequency and treatment options should be tailored on a case-by-case basis according to the clinical findings, perfusion status, extent/depth of necrosis, risk of infection, relationship with the implant, and patient characteristics. Therefore, the monitoring algorithm should be considered not as a uniform treatment regimen but as a framework that allows for re-evaluation of the dressing material, change frequency, debridement method, and, when necessary, timing of surgical intervention based on the clinical response. Within this framework, the management algorithm applied to patients with wound complications, together with an example case, is presented in Figure 1. This algorithm proposes an approach that considers surgical intervention as a last resort and places advanced wound care practices at the center of the follow-up process. In this approach, the wound care nurse and team are responsible for regular monitoring, selecting appropriate products based on wound characteristics, ensuring continuity of care, and updating the care plan based on the response [31]. There is also evidence that adequate nursing knowledge and skills regarding advanced wound care products can improve patient outcomes [32]. In this context, especially in patients who have undergone high-volume excision and are considered more at risk for perfusion, more frequent assessment in the early period and the multidisciplinary application of individualized wound care plans based on objective criteria can be considered a clinically important strategy.

## 5. Conclusions

In this retrospective cohort, a significant relationship was observed between early wound complications and excision volume after unilateral SSM and direct implant-based reconstruction. These findings indicate that the likelihood of complications increases with the volume of excision. Therefore, surgical planning and postoperative follow-up should be more clearly “risk-focused” in patients undergoing high-volume excision.

In the current study, in cases where complications developed, conservative and/or advanced wound management limited the need for re-operation to only a small number of cases. These findings support the importance of risk-based postoperative monitoring strategies and close follow-up by multidisciplinary wound care teams for patients undergoing high-volume excision. They also suggest that optimal outcomes depend on the coordinated execution of the surgeon’s perfusion-focused surgical planning and intraoperative decisions, together with early diagnosis, appropriate dressing/adjuvant treatment, and close monitoring by the wound care team.

### Limitations

The main limitations of this study were the retrospective design, relatively small sample size, and, in particular, the limited number of complications (n = 14). The absence of a control/comparison group prevents causal inferences about the effectiveness of advanced wound care, potentially preventing the demonstration of relationships with certain risk factors. Therefore, the generalizability of the results is limited, and prospective and comparative studies are needed to determine the effectiveness of advanced wound care strategies.

## Figures and Tables

**Table 1:** Demographic and clinicopathological distribution of all the patients (n = 84).

Age, years, mean ± SD	43.51 ± 6.5
Height, cm, mean ± SD	163.5 ± 5.6
Weight, kg, mean ± SD	65.9 ± 10.4
Excision volume, cm^3^, mean ± SD	521.45 ± 290.92
Smoking, n (%)	
Absent	78 (92.9)
Present	6 (7.1)
Chronic Disease, n (%)	
Absent	72 (85.7)
Hypertension	3 (3.6)
Diabetes Mellitus	1 (1.2)
Asthma	1 (1.2)
Other	8 (9.5)
Localization, n (%)	
Multicentric–multifocal	43 (51.2)
Upper outer quadrant	18 (21.4)
Upper inner quadrant	14 (16.7)
Lower inner quadrant	4 (4.8)
Central	3 (3.6)
Lower outer quadrant	2 (2.4)
Side, n (%)	
Right Breast	49 (58.3)
Left Breast	35 (41.7)
Pathology Result, n (%)	
Invasive Ductal Carcinoma	56 (66.7)
Invasive Lobular Carcinoma	14 (16.7)
DCIS	10 (11.9)
Mixed	3 (3.6)
Medullary	1 (1.2)
Axillary Procedure, n (%)	
SLNB	65 (77.3)
SLNB Axillary dissection	16 (19.0)
Axillary dissection	3 (3.6)
Neoadjuvant Chemotherapy, n (%)	
Present	30 (35.7)
Absent	54 (64.3)
Neoadjuvant Response, n (%)	
Complete Response	10 (11.9)
Partial Response	17 (20.2)
No Response	3 (3.6)
Complication, n (%)	
None	70 (84.2)
Wound dehiscence	5 (6.0)
Nipple necrosis	7 (8.4)
Infection	1 (1.2)
Hematoma	1 (1.2)
Complication-Related Re-operation, n (%)	
None	78 (93.9)
Areola excision	2 (2.4)
Wound debridement	2 (2.4)
Implant excision	2 (2.4)
Advanced Wound Care, n (%)	
Yes	10 (11.9)
No	74 (88.1)
Epithelialization time, days, mean (IQR)	53 (43)

SD, standard deviation; DCIS, ductal carcinoma in situ; SLNB, sentinel lymph node biopsy.

**Table 2:** Distribution of clinical–pathological factors according to wound complication groups.

Clinicopathological Factors	Wound Complications (−) (70 Patients, 84.2%)	Wound Complications (+) (14 Patients, 16.8%)	*p*-Value
Age (years), mean ± SD	43.7 ± 6.79	42.57 ± 5.15	*p* = 0.558 ^T^
Height, cm, median, IQR (Q1–Q3)	163 (160–167)	163.5 (155.75–168)	*p* = 0.660 ^U^
Weight, kg, mean ± SD	66.2 ± 10.76	64.79 ± 9.04	*p* = 0.647 ^T^
Excisional volume, cm^3^, median, IQR (Q1–Q3)	438.0 (269.75–662.0)	637.5 (375.5–945.5)	*p* = 0.033 ^U^
Smoking, n (%)			*p* = 0.388 ^FFH^
Absent	65 (77.3%)	12 (14.3%)	
Present	5 (6.0%)	2 (2.4%)	
Side, n (%)			*p* = 0.921 ^χ2^
Right	41 (48.9%)	8 (9.5%)	
Left	29 (34.5%)	6 (7.1%)	
Pathology Result, n (%)			*p* = 0.136 ^FFH^
Invasive ductal carcinoma	49 (58.3%)	7 (8.3%)	
Invasive lobular carcinoma	10 (12.0%)	4 (4.7%)	
DCIS	8 (9.5%)	2 (2.4%)	
Mixed	3 (3.6%)	0	
Medullary	0	1 (1.2%)	
Axillary Approach, n (%)			*p* = 0.652 ^χ2^
SLNB	55 (65.4%)	10 (12.0%)	
SLNB axillary dissection	12 (14.3%)	4 (4.7%)	
Axillary dissection	3 (3.6%)	0	
Neoadjuvant Chemotherapy, n (%)			*p* = 0.541 ^χ2^
Present	26 (30.9%)	4 (4.7%)	
Absent	44 (52.4%)	10 (12.0%)	

DCIS, ductal carcinoma in situ; SLNB, sentinel lymph node biopsy; T, Student’s *t*-test; U, Mann–Whitney U-test; χ^2^, Pearson’s chi-squared test; FFH, Fisher–Freeman–Halton Exact test; SD, standard deviation.

**Table 3:** Univariate analysis of excisional volume for wound complications.

Variables	B	O.R	95% Cl for O.R		Overall Accuracy Percentage	*p*-Value
			Lower	Upper		
Excisional volume	0.002	1.002	1.000	1.004	83.8%	0.019

**Table 4:** Wound care modality in the group with complications.

Patients	Complication	Non-Traumatic Dressing	HBOT	Topical Products	Enzymatic Debridement	Barrier Cream	Surgical Debridement	Antibiotic	Implant Excision/Areola Excision
1. Patient	Wound dehiscence	x		x		x			
2. Patient	Partial NAC necrosis	x		x	x	x	x		
3. Patient	Partial NAC necrosis	x		x	x	x	x		
4. Patient	Wound dehiscence	x		x		x			
5. Patient	Partial NAC necrosis	x		x		x			
6. Patient	Wound dehiscence	x		x		x			
7. Patient	Partial NAC necrosis	x	x	x	x	x			
8. Patient	Wound dehiscence	x	x	x		x			
9. Patient	Partial NAC necrosis	x	x	x	x	x			
10. Patient	Wound Area Infection	x		x				x	
11. Patient	Wound dehiscence	x							x
12. Patient	Hematoma								x
13. Patient	Complete NAC necrosis	x							x
14. Patient	Complete NAC necrosis	x							x

## Data Availability

The data presented in this study are available on request from the corresponding author. Data are not publicly available due to privacy concerns regarding participant confidentiality.

## References

[1] Global Cancer Observatory (GCO) Today. https://gco.iarc.fr/today/en/dataviz/pie?mode=cancer&group_populations=1&sexes=2.

[2] Benson J.R., Jatoi I., Keisch M., Esteva F.J., Makris A., Jordan V.C. (2009). Early breast cancer. Lancet.

[3] Nedeoglo E., Moog P., Jiang J., Suhova I., Machens H.G., Megerle K., Kükrek H. (2025). Trend shift from autologous to implant-based breast reconstruction. Surg. Oncol..

[4] Mota B.S., Riera R., Ricci M.D., Barrett J., de Castria T.B., Atallah Á.N., Bevilacqua J.L. (2016). Nipple- and areola-sparing mastectomy for the treatment of breast cancer. Cochrane Database Syst. Rev..

[5] Fancellu A., Houssami N., Sanna V., Porcu A., Ninniri C., Marinovich M.L. (2021). Outcomes after breast-conserving surgery or mastectomy in patients with triple-negative breast cancer: Meta-analysis. Br. J. Surg..

[6] Boneti C., Yuen J., Santiago C., Diaz Z., Robertson Y., Korourian S., Klimberg V.S. (2011). Oncologic safety of nipple skin-sparing or total skin-sparing mastectomies with immediate reconstruction. J. Am. Coll. Surg..

[7] García-Solbas S., Lorenzo-Liñán M.Á., Castro-Luna G. (2021). Long-Term Quality of Life (BREAST-Q) in Patients with Mastectomy and Breast Reconstruction. Int. J. Environ. Res. Public Health.

[8] Song Y., Wang L., Huang W., Guo S., Wu X., Zheng H. (2025). Comparison of nipple sparing and skin sparing mastectomy with immediate reconstruction based on patient reported outcomes. Sci. Rep..

[9] Romanoff A., Zabor E.C., Stempel M., Sacchini V., Pusic A., Morrow M. (2018). A Comparison of Patient-Reported Outcomes After Nipple-Sparing Mastectomy and Conventional Mastectomy with Reconstruction. Ann. Surg. Oncol..

[10] Bryan J.L., Ockerman K.M., Spiguel L.R., Cox E.A., Han S.H., Trieu N., Blondin-Fernandez M., Heath F., Sorice-Virk S. (2024). Postoperative complications of direct-to-implant and two-staged breast reconstruction: A stratified analysis. Plast. Surg..

[11] Gschwantler-Kaulich D., Leser C., Salama M., Singer C.F. (2018). Direct-to-implant breast reconstruction: Higher complication rate vs cosmetic benefits. Breast J..

[12] Gulcelik M.A., Dogan L., Karanlik H., Akinci M., Ugurlu M.U., Gulluoglu B.M. (2025). Profile of surgical complications and complication-led reoperation rates in breast cancer patients who underwent oncoplastic breast surgery with volume displacements. Eur. J. Surg. Oncol..

[13] Koc B.I., Morkavuk S.B., Akyuz S., Aygun G., Ozdemir O., Gulcelik M.A. (2025). Postoperative outcomes of one-step implant-based breast and ovarian surgery in high-penetrance gene mutation: A single-center experience. J. Clin. Med..

[14] Bogdan R.G., Helgiu A., Cimpean A.M., Ichim C., Todor S.B., Iliescu-Glaja M., Bodea I.C., Crainiceanu Z.P. (2024). Assessing fat grafting in breast surgery: A narrative review of evaluation techniques. J. Clin. Med..

[15] Lemaine V., Hoskin T.L., Farley D.R., Grant C.S., Boughey J.C., Torstenson T.A., Jacobson S.R., Jakub J.W., Degnim A.C. (2015). Introducing the SKIN score: A validated scoring system to assess severity of mastectomy skin flap necrosis. Ann. Surg. Oncol..

[16] National Healthcare Safety Network (NHSN) Surgical Site Infection (SSI) Event. Centers for Disease Control and Prevention (CDC): January 2026. https://www.cdc.gov/nhsn/pdfs/pscmanual/9pscssicurrent.pdf.

[17] Krekel N.M., Zonderhuis B.M., Stockmann H.B., Schreurs W.H., van der Veen H., de Lange de Klerk E.S., Meijer S., van den Tol M.P. (2011). A comparison of three methods for nonpalpable breast cancer excision. Eur. J. Surg. Oncol..

[18] Haloua M.H., Volders J.H., Krekel N.M., Barbé E., Sietses C., Jóźwiak K., Meijer S., van den Tol M.P. (2016). A nationwide pathology study on surgical margins and excision volumes after breast-conserving surgery: There is still much to be gained. Breast.

[19] Zarei M., Carlson G.W. (2024). Periareolar skin-sparing mastectomy and immediate implant-based reconstruction: A reappraisal. Ann. Plast. Surg..

[20] Corban J., Shash H., Safran T., Sheppard-Jones N., Fouda-Neel O. (2017). A systematic review of complications associated with direct implants vs. tissue expanders following Wise pattern skin-sparing mastectomy. J. Plast. Reconstr. Aesthetic Surg..

[21] Clarijs M.E., Peeters N.J.V., van Dongen S.A., Koppert L.B., Pusic A.L., Mureau M.A., Rijken B.F. (2023). Quality of life and complications after nipple-versus skin-sparing mastectomy followed by immediate breast reconstruction: A systematic review and meta-analysis. Plast. Reconstr. Surg..

[22] Paprottka F.J., Schlett C.L., Luketina R., Paprottka K., Klimas D., Radtke C., Hebebrand D. (2019). Risk Factors for Complications after Skin-Sparing and Nipple-Sparing Mastectomy. Breast Care.

[23] Wengler C.A., Valente S.A., Al-Hilli Z., Woody N.M., Muntean J.H., Abraham J., Tendulkar R.D., Djohan R., O’Rourke C., Crowe J.P. (2017). Determinants of short and long term outcomes in patients undergoing immediate breast reconstruction following neoadjuvant chemotherapy. J. Surg. Oncol..

[24] Ito H., Ueno T., Suga H., Shiraishi T., Isaka H., Imi K., Imoto S. (2019). Risk factors for skin flap necrosis in breast cancer patients treated with mastectomy followed by immediate breast reconstruction. World J. Surg..

[25] Nguyen C.L., Zhou M., Easwaralingam N., Seah J.L., Chan B., Graham S., Azimi F., Mak C., Pulitano C., Warrier S. (2026). Mastectomy Skin Flap Necrosis after Implant-Based Breast Reconstruction: Intraoperative Predictors and Indocyanine Green Angiography. Plast. Reconstr. Surg..

[26] Seth I., Xie Y., Lim B., Rozen W.M., Hunter-Smith D.J. (2024). Defining skin-sparing mastectomy surgical techniques: A narrative review. Ann. Breast Surg..

[27] Verras G.I., Mulita F. (2024). Butyrylcholinesterase levels correlate with surgical site infection risk and severity after colorectal surgery: A prospective single-center study. Front. Surg..

[28] Lindley L.E., Stojadinovic O., Pastar I., Tomic-Canic M. (2016). Biology and Biomarkers for Wound Healing. Plast. Reconstr. Surg..

[29] O’Connell R.L., Rusby J.E. (2015). Anatomy relevant to conservative mastectomy. Gland Surg..

[30] Santanelli F., Longo B., Sorotos M., Farcomeni A., Paolini G. (2013). Flap survival of skin-sparing mastectomy type IV: A retrospective cohort study of 75 consecutive cases. Ann. Surg. Oncol..

[31] Acar K., Aygin D. (2015). Yaşlılarda yara gelişimi risk faktörleri, önleme ve bakım yaklaşımları. Yoğun Bakım Hemşireliği Derg..

[32] Öztürk D., Karadağ A. (2019). Stoma ve Yara Bakım Hemşireliği’nin tarihsel gelişim süreci: Türkiye örneği. Hemşirelikte Eğitim ve Araştırma Derg..

